# Complex Lifestyle and Psychological Intervention in Knee Osteoarthritis: Scoping Review of Randomized Controlled Trials

**DOI:** 10.3390/ijerph182312757

**Published:** 2021-12-03

**Authors:** Bryan Yijia Tan, Tivona Thach, Yasmin Lynda Munro, Soren Thorgaard Skou, Julian Thumboo, Josip Car, Lorainne Tudor Car

**Affiliations:** 1 Department of Orthopaedic Surgery, Woodlands Health Campus, Singapore 768024, Singapore; 2 Lee Kong Chian School of Medicine, Nanyang Technological University, Singapore 308232, Singapore; yasminmunro@ntu.edu.sg (Y.L.M.); josip.car@ntu.edu.sg (J.C.); lorainne.tudor.car@ntu.edu.sg (L.T.C.); 3 Faculty of Medicine, University of New South Wales, Sydney, NSW 2052, Australia; z5184040@unsw.edu.au; 4 Research Unit for Musculoskeletal Function and Physiotherapy, Department of Sports Science and Clinical Biomechanics, University of Southern Denmark, 5230 Odense, Denmark; stskou@health.sdu.dk; 5 Department of Physiotherapy and Occupational Therapy, Næstved-Slagelse-Ringsted Hospitals, 4200 Slagelse, Denmark; 6 Department of Rheumatology and Immunology, Singapore General Hospital, Singapore 169608, Singapore; julian.thumboo@singhealth.com.sg

**Keywords:** knee osteoarthritis, randomized controlled trials, intervention, lifestyle, psychological, psychosocial, scoping review

## Abstract

Knee osteoarthritis (OA) causes pain, disability and poor quality of life in the elderly. The primary aim was to identify and map out the current evidence for randomised controlled trials (RCTs) on complex lifestyle and psychosocial interventions for knee OA. The secondary aim was to outline different components of complex lifestyle and psychosocial interventions. Our scoping review searched five databases from 2000 to 2021 where complex lifestyle or psychosocial interventions for patients with knee OA were compared to other interventions. Screening and data extraction were performed by two review authors independently and discrepancies resolved through consensus and in parallel with a third reviewer. A total of 38 articles were selected: 9 studied the effectiveness of psychological interventions; 11 were on self-management and lifestyle interventions; 18 looked at multifaceted interventions. This review highlights the substantial variation in knee OA interventions and the overall lack of quality in the current literature. Potential areas of future research, including identifying prognostic social factors, stratified care models, transdisciplinary care delivery and technology augmented interventions, have been identified. Further high-quality RCTs utilizing process evaluations and economic evaluation in accordance with the MRC guidelines are critical for the development of evidence-based knee OA programs globally.

## 1. Background

Osteoarthritis (OA) is among the largest and fastest growing cause of pain, disability and poor quality of life in the elderly around the world [1]. International guidelines are all consistent in their recommendations of lifestyle changes, with exercise and weight loss programs recommended as the first-line treatments of knee OA with a stepwise approach, where surgery is considered only when non-surgical treatment fails [2,3]. However, international studies report that at least 60% of patients from established healthcare systems around the world such as Australia, Canada and the US are not receiving optimal conservative treatment [4,5]. In order to tackle the growing OA epidemic, many complex multidisciplinary programs have been developed around the world aiming to enhance care coordination and improve outcomes [6,7].

A review article on the challenges and controversies of complex interventions in OA highlighted self-management and lifestyle modification as a particular area of complexity given the large variation in delivery and content [7]. A systematic review on self-management educational programs in OA outlined the overall lack of quality in this area and the heterogeneity of intervention, ranging from skill/technique acquisition and health-directed activity to self-monitoring and insight [8]. In addition, with the move away from the traditional biomedical model, which has been proven to be inadequate with poor concordance between pain, disability and pathology [9], towards a biopsychosocial model [10], there has been a greater recognition of the psychosocial factors in knee OA [11,12]. As a result, the use of psychosocial interventions in the management of OA has gained popularity over the past two decades with many multidisciplinary knee OA programs now including psychosocial elements in combination with lifestyle interventions, augmenting traditional pharmacological and surgical treatments [13]. A substantial variation exists in the delivery of such programs including the mode (face-to-face, telephone, internet), audience (group, individual), duration, frequency and personnel delivering the intervention.

There exists a lack of evidence: in particular, the lack of gold standard high-quality randomized controlled trials (RCTs) to inform efficacy and effectiveness of these complex psychosocial and lifestyle programs in knee OA, and regarding psychosocial interventions, which previous reviews do not address. Of note, moving beyond just high-quality evidence to implementation is critical to facilitate translation of evidence-based based research into routine clinical practice. The Medical Research Council (MRC) guidelines for the development and evaluation of complex interventions recommend the incorporation of a process and economic evaluation to the main trial to aid eventual implementation [14]. A scoping review was undertaken with the primary aim of identifying and mapping RCTs, including the presence or absence of process and economic evaluations on complex lifestyle and psychosocial interventions for knee OA, in order to delineate potential gaps in the evidence to guide future research in this area [15]. This is in line with other scoping review focusing on RCTs that have been conducted previously [16,17]. The secondary aim was to outline different components of complex lifestyle and psychosocial interventions from the interventions identified.

## 2. Methodology

We used the Joanna Briggs Institute scoping review methodology developed in 2016 by Peters et al. [18]. Extension of the PRISMA-ScR guidelines for scoping reviews was used to ensure robust methodology and reporting of results [19].

### 2.1. Study Inclusion Criteria and Selection

The PICOTS framework was used to specify inclusion and exclusion criteria for this review (Table 1) [20]. We included RCTs in which a complex lifestyle or psychosocial intervention for adults more than 18 years old with knee OA was compared to other forms of intervention such as a different form of complex intervention, usual care, uni-disciplinary approach, placebo intervention or no intervention. We excluded studies in which only a single intervention was considered. For example, pure exercise intervention with no self-management or education component. We also excluded studies that did not address knee OA specifically, such as other joint arthritis, inflammatory arthritis and those including surgical interventions.

Complex interventions have several definitions. For the purposes of this scoping review, the Agency for Healthcare Research and Quality (AHRQ) Evidence-based Practice Centre Program definition for complex intervention was used [21]. Complex intervention must contain two essential characteristics, namely multiple components (intervention complexity) and complicated/multiple causal pathways, feedback loops, synergies, and/or mediators and moderators of effect (pathway complexity). Other additional optional components include multiple participants/group/organizational levels (population complexity), multifaceted adoption/uptake/integration strategies (implementation complexity) and dynamic multidimensional environment (contextual complexity).

Exercise or physical based intervention, if done in combination with other treatment modalities such as education, is considered a complex intervention as there is variation in terms of delivery and adherence. Psychosocial interventions are defined as any intervention that emphasizes psychological or social factors rather than biological factors. This definition allows for the inclusion of psychological interventions and health education, as well as interventions with a focus on social aspects, such as social support [22]. Specifically, psychological intervention is defined based on the content, proposed mechanism or method of delivery [23]. Firstly, the intervention is anchored by established psychological theory such as cognitive behavioural therapy (CBT) and secondly, the intervention is delivered by a psychologist or a healthcare professional who has received transdisciplinary training in psychological interventions. Even if the psychological intervention is an “isolated” intervention, it is deemed to be complex due to the multiple approaches (intervention complexity) and moderators of effect (pathways complexity) that are part of any psychological intervention. This same rationale applies to “isolated” lifestyle interventions.

Pharmacological and surgical related interventions were excluded for this review as the focus on this review is lifestyle and psychosocial interventions, which predominantly focus on behavioural change and other self-management strategies rather than pharmacological or surgical procedures. Similarly, interventions that target post-surgical population was also be excluded.

All the articles were screened independently by two authors (BT and TT) in three consecutive rounds: based on (1) title (2) abstract to exclude articles that were obviously not within the scope of topic. Subsequently, the remaining articles were screened by (3) full manuscript text to determine eligibility based on the inclusion and exclusion criteria. Any disagreements were resolved after discussion between both authors in conjunction with a third review author in a consensus meeting (LTC).

### 2.2. Search Methods for Identification of Studies

A literature search was conducted using a search strategy. Keywords were developed based on a broad exploration of the topic with an information specialist and reviewing previous search strategies for systematic and scoping reviews that were conducted on similar topics previously [24,25,26,27,28]. Truncations were used to search for variant forms of the keywords. Boolean Operators such as “and” and “or” were used to yield more specific search results. We limited our searches to RCTs only using pre-defined RCT filters, with the publication date from the year 2000 to 15 February 2021, to ensure the latest evidence would be appraised as part of this scoping review, as knee OA interventions and delivery models have changed substantially over time. Only articles published in English were included.

On 15 February 2021, we searched the following five electronic databases: Ovid MEDLINE^®^ and Epub Ahead of Print, In-Process & Other Non-Indexed Citations, Daily and Versions(R); Embase; Cochrane Central Register of Controlled Trials (CENTRAL) (Wiley); PsycInfo (EBSCOHost); CINAHL (EBSCOHost). The respective search strategies are included as Supplementary S1.

### 2.3. Data Extraction and Quality Assessment

The data extraction form was created and piloted by the two authors (BT and TT) to systematically record study characteristics. It was subsequently adjusted to ensure that all relevant information was included in the final data extraction sheet. Relevant data such as study population, intervention details and key findings were independently extracted by each author in the standard form and summarized into a table format. The reporting of interventions in the included studies was assessed using the Template for Intervention Description and Replication (TIDieR) template, which assesses the completeness and comprehensiveness of the intervention description and reporting [29]. The TIDieR template consists of 12 components including the providers, intervention delivery details and fidelity. Each of the included studies were given a score based on the percentage of components that were reported. In addition, the presence of an accompanying economic evaluation or process evaluation to the RCT were noted as part of the review regardless of whether it was part of the same publication or published separately. It was also noted if the study had a prior published study protocol, trial registration and were reported in accordance with the CONSORT guidelines [30]. The data extraction form was filled by the two authors (BT and TT) with any discrepancies resolved in a consensus meeting.

### 2.4. Data Synthesis

Consistent with the scoping review methodology, the focus was on the extent and nature of the available evidence and not on the quality of the evidence. A limited evaluation of the quality of the evidence, particularly with the adherence of the TIDieR template, was performed. The data were presented through a narrative review, grouping the interventions into 3 broad categories (psychological interventions, lifestyle and self-management interventions and multifaceted) and use of frequency analysis and trend analysis to chart the classified results.

## 3. Results

**Figure 1:**
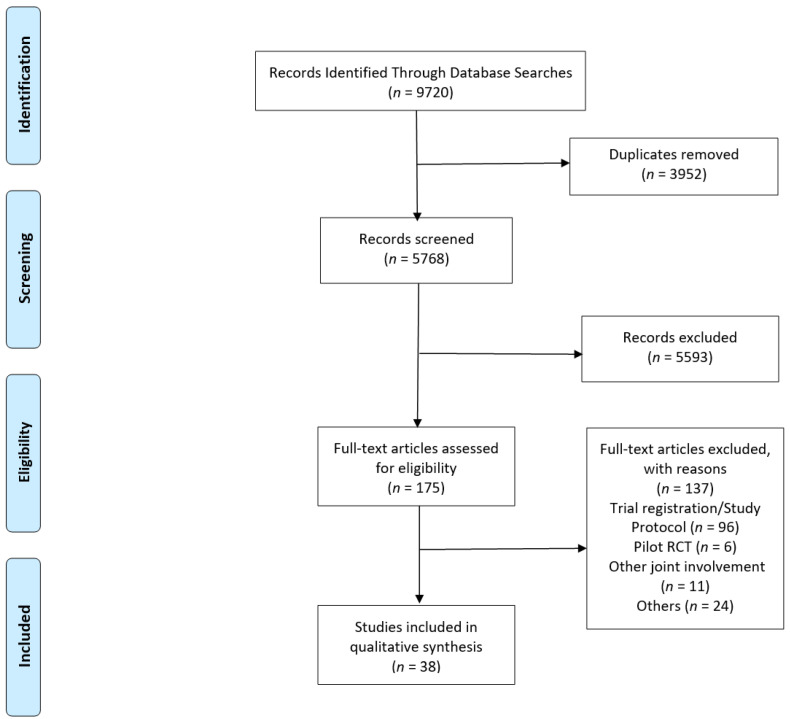
PRISMA flowchart.

The literature search yielded a total of 5768 results after duplicates were removed. After title and abstract review screening, 175 articles were selected for a full manuscript review. A total of 38 articles were selected for this scoping review. The PRISMA flow diagram has been included for reference (Figure 1).

### 3.1. Categories

Results were grouped into four broad categories based on the intervention studied in the trial (psychological, self-management/lifestyle, nutrition-based and exercise and education). A summary is provided in Table 2.

#### 3.1.1. Psychological Intervention

Our search yielded nine studies that involved a predominant psychological intervention. Four studies were isolated psychological interventions, while five studies looked at psychological interventions as part of a combination treatment. Two of the four isolated psychological interventions focused on patients with knee OA but with a concurrent psychological condition such as insomnia and depression [31,32]. Hausmann et al., 2018, investigated the effectiveness of a positive psychological skill-building intervention program with veterans [33] and Helminen et al., 2015, looked at group CBT for rehabilitation in knee OA [34].

Combination interventions generally involved the addition of an exercise component in addition to the psychological elements [35,36]. Bennell et al. studied the effectiveness of pain coping skills training (PCST) in two different delivery formats with exercise. Firstly, PCST and exercise delivered through physiotherapists who had received appropriate training by psychologists [37] and, secondly, PCST and exercise by physiotherapists but delivered through internet delivery instead [38]. Ahn et al., 2020, described a program based on the Interactive Model of Client Health Behaviour (ICMCH) focusing on components of client singularity (cognitive–affective–behavioural skills), client–professional interaction and patient activation in a combination of individual (home or telephonic) and group sessions delivered by community health nurse practitioners (CHNPs) who had received prior training [39].

#### 3.1.2. Self-Management and Lifestyle Intervention

A total of 11 studies were identified that studied self-management and lifestyle intervention and did not involve any supervised exercise/nutritional/psychological components [40,41,42,43,44,45]. These programs were conducted on an individual level, group level or in combination. They were delivered by a variety of professionals including nurses, doctors, physiotherapist and non-professional groups, e.g., lifestyle counsellors. Components of the education behavioural change programs generally consisted of exercise, nutrition, joint protection, shoe wear, assistive device use and pain management.

Two of the programs were delivered digitally. Mecklenburg et al., 2018, described the Hinge Health Digital Program, which was delivered through tablet computers and digital sensor-guided exercises through the Hinge Health application supported by personal coaching [46]. Allen et al., 2018, described the IBET program, which utilized a tailored algorithm for exercise progression based on individual functional levels. Video display, automated reminders and progression tracking were part of the IBET program [47]. Three programs were delivered telephonically [48,49,50].

#### 3.1.3. Exercise and Education, Nutrition-Based Interventions

Eleven studies focused on interventions primarily targeting supervised exercise with variations of education or self-management programs [51,52,53,54,55,56,57,58,59,60,61,62]. Physical exercise generally consisted of a combination of strength, resistance and aerobic exercises usually delivered by a trained physiotherapist, with two studies looking at the effectiveness of exercise implemented through community walking programs [51,52]. Education components typically focused on understanding the condition and self-efficacy strategies to promote effective positive behavioural change. These sessions were delivered either to individuals, groups or in combination. Educators included health coaches, nurses or allied health professionals. Several programs included telephonic follow up. Five studies had a core nutrition component with a dietician or nutritionist, with elements of exercise and education that focused specifically on overweight patients [63,64,65,66,67].

### 3.2. Interventions Overview and Combination Mapping

**Figure 2:**
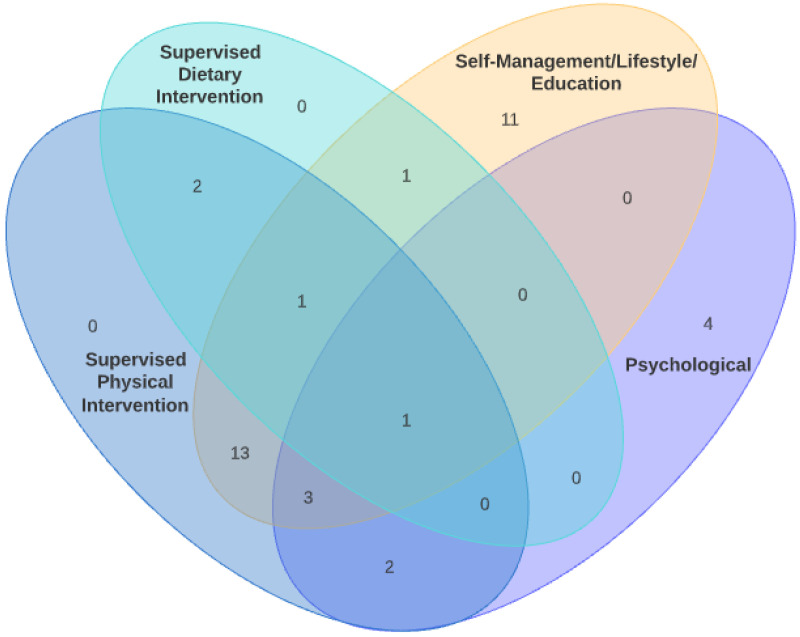
Venn diagram of the different intervention combinations.

The trials were mapped according to four domains of interventions that were identified as part of this review. The four domains identified were supervised physical/exercise-based, supervised nutritional/dietary-based, psychological and self-management/lifestyle interventions. While some interventions occurred in isolation, many of the interventions were delivered in combination. If the intervention involved any form of physical program but the participants were taught to do the prescribed exercises at home as part of an overall lifestyle change effort, it was deemed to be in the self-management/lifestyle domain. The most frequent combination of interventions was a combined self-management/lifestyle and supervised physical intervention that occurred in 13 studies. The next most frequent intervention was a pure self-management/lifestyle program with 10 studies. Figure 2 illustrates the evidence map visually through a Venn diagram on the different combinations. An overview and summary of the various interventions in terms of the *what* (content), *who* (providers), *how* (delivery format), *where* (setting) and *when* (timing) is provided in Table 3.

### 3.3. Interventions That Incorporated Social Support (Peers, Family, Spouse, Coach)

While many interventions incorporated a group delivery format, only a handful of interventions specifically mentioned improving social support or relationships as one of the core strategies. Somers et al., 2012, looked at pain coping skills training (PCST) with an emphasis on **relationship support** under the LEARN (lifestyle, exercise, attitudes, relationships and nutrition) framework [36]. The IDEA trial by Messier at al., 2013, studied the effectiveness of combination treatment but with strategies for improved adherence built into the program through the application of a behavioural toolbox, counselling, **social support** and incentives [66]. Mecklenburg et al., 2018, described the Hinge Health Digital Program, which was delivered through tablet computers and digital sensors through the Hinge Health application. It focused on digital sensor-guided exercise therapy, weight loss and **psychosocial support** through a personal coach [46].

Two studies described the implementation of a walking program coupled with an educational component. Brosseau et al., 2012, studied the effectiveness of a supervised community-based aerobic walking problem (SCAWP), in addition to a behavioural intervention through the use of coaching, goal setting and social support [51]. Wallis et al., 2017, included a prescribed walking program in conjunction with regular physiotherapy and education [52]. Participants in the trial were encouraged to engage in **social supports** such as walking with a friend, family member or other research participants. Focusing specifically on **spousal support**, Keefe et al., 2004, investigated the effectiveness of spouse-assisted pain coping skill training (SA-CST) and exercise training [35].

### 3.4. Quality of Study Methodology and Reporting

There was a wide variation in the quality of the RCTs conducted. Some of the RCTs had a well-described study protocol, trial registration, had an adequate sample size and were reported in line with the CONSORT guidelines [30], while other studies were lacking in some of these areas. Sample sizes ranged from as low as 46 to as high as 2203 subjects. Specifically focusing on the quality of intervention description reporting based on the TIDieR template (12 components), the mean score was 72%, with some of the studies scoring as low as 58%. The scores based on the TIDieR template are included in Supplementary S2.

### 3.5. Implementation Components in the Study Design

A total of 19 studies out of the 38 studies (50.0%) included some form of process evaluation. These process evaluations mainly focused simply on patient’s adherence through the use of questionnaires, exercise logs and attendance frequency. None of the studies undertook a full process evaluation with patient, healthcare professionals or stakeholder engagement through qualitative methods using evaluation or established implementation frameworks such as RE-AIM [68]. Only four studies (10.5%) had an accompanying economic evaluation.

### 3.6. Summary

A summary of all the included studies, with a breakdown of the individual intervention, outcome measures, study size, results based on primary outcome and presence of a process or economic evaluation, is included in Supplementary S2.

## 4. Discussion

### 4.1. Wide Variation in Interventions

This scoping review highlights a wide diversity in this area of research. Previous reviews on OA programs have highlighted the poor descriptions of programs and, in particular, the need for precise definitions and details of the theoretical underpinnings, components and mechanisms for such programs [26]. Our review has demonstrated the significant variations in the content delivered, either in isolation or in various combinations. Beyond the content variation, there exist significant variations in the treatment providers, delivery formats, settings and timings, which are summarized in Table 3. Table 3 provides a broad and comprehensive overview of the various options in all of the key domains in program or intervention development that have been conducted in lifestyle and psychosocial interventions in knee OA. This can aid researchers and program designers in the development and design of future complex lifestyle and psychosocial interventions targeting specifically knee OA and, potentially, other similar chronic musculoskeletal degenerative conditions.

### 4.2. Quality of Study Methodology and Reporting

There was a wide variation in the quality of the RCTs in terms of the sample sizes, power and reporting quality. In particular, it was noted that the majority of the studies did not fully describe their intervention based on the TIDieR template, which was introduced to provide a standardized format for researchers to follow [29]. Complete and clear description of an intervention allows for other researchers to replicate or build on the research findings, which is particularly critical in complex interventions.

### 4.3. Moving from Evidence to Implementation

In the studies identified in this review, only a handful of them included any sort of process evaluation or economic evaluation. The MRC guidelines for the development and evaluation of complex interventions recommend a series of key steps ranging from feasibility or pilot testing to development, evaluation and implementation [14]. In addition to the usual primary and secondary quantitative outcome measures, incorporating a process evaluation and economic evaluation is strongly recommended to aid the implementation and translation of the complex intervention into routine clinical practice. Process evaluations focus on key concepts such as fidelity, casual mechanisms and contextual factors through mixed methods approaches [69], while economic evaluation assesses the cost-effectiveness and overall financial sustainability for any intervention. These would help avoid the “valley of death” that is often seen where good evidence does not get translated into actionable policy or clinical practice change [70].

Moving forward, more high-quality, adequately powered and well-reported RCTs, incorporating key elements of process evaluation and economic evaluation are critical. There has been a promising trend towards effectiveness–implementation hybrid trials that incorporate both effectiveness and implementation components such as a process evaluation through mixed method approaches in the study design [71]. Such studies would not only add meaningfully to the existing body of evidence but, more importantly, would allow translation and implementation into clinical practice.

### 4.4. Future Directions for Lifestyle and Psychosocial Intervention Development

#### 4.4.1. Social Factors and Social Interventions in Knee OA Outcomes

Research has shown the significant social factors that have been identified, including social position such as socioeconomical status, built environment, environmental exposure, occupational factors, health literacy and social support/coping resources, in mediating osteoarthritis outcomes [72]. Social interventions are defined as interventions that are intentionally implemented change strategies that aim to impede or eradicate risk factors, activate and/or mobilize protective factors, reduce or eradicate harm, or introduce betterment beyond harm eradication; thus, social interventions encompasses a range of psychotherapies, treatments and programs [73].

While many such factors are at a systems level, such as built environment, or non-modifiable factors such as socioeconomical status, there are several individual modifiable factors such as social support that can be targeted by social interventions. We found several studies that described the use of improving health literacy through education and establishing peer/family/spousal support as a strategy to improve intervention compliance and adherence. A review on social support as a factor in OA management programs described two broad categories in this field. Firstly, social interaction among peers in group-based interventions and, secondly, emotional/informational support received from healthcare professionals [26]. However, the review also noted that theoretical underpinning and precise definitions were lacking. Understanding and exploring the complex relationship social factors have with clinical outcomes is an area of future research in order to develop more holistic and comprehensive patient-centred psychosocial and lifestyle interventions in knee OA.

#### 4.4.2. Stratified Care Model vs. Stepwise Care Model

Traditionally, OA treatment has generally followed a stepwise approach [74] consistent with what this review has shown. In contrast, taking a tailored personalized approach may be an alternate way forward in order for patients to have the right treatment in a timely manner through the use of appropriate early triaging. The ideal model of care is one where patients have the “*Right care*, delivered at the *right time*, by the *right team*, in the *right place*, with the *right resources*” [75]. This approach has been shown to be highly effective in other musculoskeletal conditions, such as low back pain, with the use of the STarT Back Tool [76].

Particularly in the area of lifestyle self-management and psychosocial intervention, similar tailoring and stratification of treatment could be conducted. Patients with knee OA are a very heterogenous population. Gaining a deeper understanding of this population and identifying appropriate prognostic factors, particularly psychosocial factors, using the biopsychosocial framework and the development of validated triaging tools, is a key area for research moving forward. Several triaging tools such as the modified STarT Back Tool [77] and STarT MSK tool [78] developed from the original STarT Back Tool, which incorporate a significant psychological component, have shown significant promise but, at the current time, are still not fully validated for use in routine clinical practice.

#### 4.4.3. Looking beyond Traditional Roles: Transdisciplinary Training

Several studies in our review demonstrated the effectiveness of psychological interventions that were not delivered by a clinical psychologist but by other healthcare professionals who had been given prior training in psychological intervention. This is an area of great promise that suggests that such skills are potentially transferable to other healthcare professional groups, and transdisciplinary training can be provided to allow for more comprehensive care to be delivered by a smaller core group of healthcare professionals. Looking beyond that, several studies utilized a health or lifestyle coach in place of a traditional healthcare professional, suggesting the possibility of certain lifestyle and psychosocial interventions being delivered by staff outside the traditional healthcare professional circle.

#### 4.4.4. Treatment Delivery Evolution: Digital and Technology Enabled Interventions

Based on this review, the first RCTs incorporating digital and technology-enabled intervention as part of a complex lifestyle or psychosocial intervention for knee OA were published in 2017 onwards and, since then, the number of studies has been growing steadily. As technology becomes part and parcel of our everyday life, there is a clear trend that this evolution is taking place in the realm of lifestyle and psychosocial interventions in knee OA. This review demonstrated the scope of technology, ranging from the incorporation of technology such as health apps and video display as a means for intervention delivery augmentation of traditional face-to-face interactions to the use of wearable technology to monitor activity level and treatment compliance as part of a lifestyle change and self-management program. This is in line with other similar studies on this topic [79]. In light of the current COVID-19 pandemic with social distancing becoming the “new normal”, the continued development of such digital and technological-based intervention is key is ensuring that patients with knee OA receive effective and timely treatment, augmenting traditional treatment delivery models.

### 4.5. Strength and Limitations

This is the first review mapping out the current evidence on complex lifestyle and psychosocial interventions for knee OA. The review presents a broad overview of the current evidence and, more critically, highlights the lack of high-quality evidence in this area and gaps in literature that will provide direction for future research.

Secondly, the different and varied components (content, providers, delivery format, location and time) of complex lifestyle and psychosocial interventions have been summarized in an easily accessible format that can potentially be used to aid researchers and program designers in the development and design of future complex lifestyle and psychosocial interventions in knee OA and, potentially, other musculoskeletal conditions.

There are inherent limitations to a scoping review compared to a systematic review and meta-analysis. The quality of evidence is not appraised as part of this review. In addition, our review does not address the effectiveness of the included interventions. As pointed out in the results, there was a wide variation in the quality of the RCT and heterogeneity of the included studies. As such, no definitive conclusions can be made with regards to the relative effectiveness of the different interventions. In addition, the non-English literature and other experimental study designs were not included that could potentially have provided a more comprehensive picture of the available evidence.

## 5. Conclusions

The scoping review maps out the current literature available on complex lifestyle and psychosocial interventions in knee OA, highlighting the substantial variation in interventions and overall lack of reporting quality in this area. Future research should focus on the impact of social factors, stratified care models, exploring transdisciplinary training and technology-augmented interventions. Further high-quality RCTs utilizing a thorough development process, process evaluation and economic evaluation in accordance with the MRC guidelines are needed to critically inform the development of evidence-based knee OA programs around the world.

## Figures and Tables

**Table 1:** Inclusion and Exclusion Criteria.

PICOTS Framework Components	Inclusion Criteria	Exclusion Criteria
Population	Adults more than 18 years old with knee osteoarthritis, chronic knee pain	Other joint arthritis Inflammatory arthritis Surgical intervention
Intervention	Complex lifestyle or psychosocial intervention	Surgical intervention Single intervention Pharmacological interventions
Comparison	Usual care, single intervention, placebo, another form of complex intervention, no intervention	No control arm
Outcome	Any outcome measures	Nil
Timing	Any duration	Nil
Setting	Any setting, e.g., hospital, outpatient clinic, community	Nil
Design	Randomized Control Trials	Non-randomized controlled trials Other study designs

**Table 2:** Summary of Results.

Categories	Number of Studies
Psychological Interventions	Isolated Psychological Intervention (4 studies) Psychological Intervention as part of combination treatment (5 studies)
Self-management and lifestyle Interventions	Non-digital Interventions (6 studies) Digital (2 studies) and Telephonic-based Intervention (3 studies)
Exercise and Education Interventions	Supervised Exercise and Education (9 studies) Community Walking Programs (2 studies)
Nutrition-based Interventions (Overweight patients)	Dietician or nutritionist-led weight loss and exercise programs (5 studies)

**Table 3:** Summary of various interventions.

**Content**	Physical, e.g., *Muscle strengthening, walking exercise, balance/proprioception, spa sessions* Dietary, e.g., *Dietitian/nutritionist sessions, food diary* Education, e.g., *Health education (lectures), self-management skills* Psychological, e.g., *Counselling, psychologist-led sessions, cognitive behavioral therapy, pain coping skills* Multi-modal approach Tailored or stratified approach Adherence strategy, e.g., *Logbooks, telephone calls, lesson completion questionnaires, exercise diaries, weight assessments, incentives*
**Setting**	Home Community, e.g., Community centre Healthcare institution, e.g., *Hospital, exercise rehabilitation facility*
**Timing**	Duration and frequency of sessions Total duration of program Phased approach, e.g., *Initial intensive phase followed by maintenance phase*
**Provider**	Healthcare professionals, e.g., *Doctors, nurses, pharmacists, allied health (physiotherapists, occupational therapists, dietitians/nutritionists/psychologists)* Non-healthcare professionals, e.g., *Health coach, lifestyle counsellor*
**Delivery Format**	Face-to-face Individual or group based Internet/digital, e.g., *Teleconference for class/course conduct, telerehabilitation, Sensory Motion Technology, health apps* Written material, e.g., *Information pamphlet, log books, newsletter* Home-based, self-directed program, e.g., *Home exercise schedule, online course*

## Data Availability

The data presented in this study are available upon request from the corresponding author.

## Supplementary Materials

The following are available online at https://www.mdpi.com/article/10.3390/ijerph182312757/s1. Supplementary S1: Search Strategies. Supplementary S2: Summary of Included Studies.
