# Supplementary material for: Complex Lifestyle and Psychological Intervention in Knee Osteoarthritis: Scoping Review of Randomized Controlled Trials
Source: Int J Environ Res Public Health. 2021 Dec 3;18(23):12757. doi: 10.3390/ijerph182312757 (PMC8657138; doi:10.3390/ijerph182312757)
Supplement: Supplementary file 1 [file ijerph-18-12757-s001.zip › ijerph-1403346-supplementary/IJERPH Supplementary S1.pdf]

Supplementary S1

1) Medline (Ovid) Search Strategy

Database: Ovid MEDLINE(R) and Epub Ahead of Print, In-Process & Other Non-Indexed Citations, Daily and Versions(R) <1946 to February 15, 2021>

Search Strategy:

----

1 Osteoarthritis, Knee/ or Knee Joint/
2 ("Knee Pain" or "Osteoarthritis Of Knee" or "knee Osteoarthritis" or "knee OA").ab,ti,tw.
3 (knee? adj (arthritis or osteoarthritis* or degeneration* or disease? or pain?)).ab,ti,tw.
4 ((radiographic* or symptomatic* or clinical*) adj1 knee osteoarthritis*).ab,ti,tw.
5 1 or 2 or 3 or 4
6 Exercise/ or Physical Exertion/ or Physical Fitness/ or "Physical Education and Training"/ or Sports/ or exp Exercise Therapy/ or exp Occupational Therapy/ or exp "Physical Therapy (Specialty)"/
7 (Physical* adj (fit or fitness or activit* or training? or therapy or therapies or endur* or life-style or lifestyle)).ab,ti,tw.
8 (exercise? or rehabilitation* or treatment* or sport* or walk* or bicycl* or (activ* adj (lifestyle or life-style))).ab,ti,tw.
9 ((resist* adj (exercise? or therapy or therapies or training?)) or occupational therap* or physiotherap*).ab,ti,tw.
10 6 or 7 or 8 or 9
11 Weight Loss/ or Body Weight/ or exp Energy Intake/ or exp Overweight/ or Diet/ or Diet, Vegetarian/ or Diet, Carbohydrate-Restricted/ or Diet Therapy/ or Diet, Reducing/
12 (dietary restriction? or meal replacement? or weight loss or intentional weight loss or hypocloric diet or obesity).ab,ti,tw.
13 (diet adj2 (therapy or therapies or treatment?)).ab,ti,tw.
14 11 or 12 or 13
15 Psychotherapy/ or psychology/ or psychology, educational/ or psychology, social/ or Cognitive Behavioral Therapy/ or Relaxation Therapy/ or Relaxation/ or "Imagery (Psychotherapy)"/ or Hypnosis/ or exp Behavior Therapy/
16 (psychotherap* or psychological or psychology or psycho-education or cognitive therap* or behavio?ral therap* or relaxation or imagery or hypnosis or psychosocial or (adapt* adj behavio?r?) or (behavio?r? adj (therap* or intervention*))).ab,ti,tw.
17 15 or 16
18 Health Education/ or Patient Education as Topic/ or Health Promotion/ or Primary Prevention/
19 (promot* or educat* or program? or programme?).ab,ti,tw.
20 18 or 19
21 (multidisciplinar* or interdisciplinar* or multiprofessional* or multimodal*).ab,ti,tw.
22 14 or 17 or 20 or 21
23 5 and 10 and 22
24 randomized controlled trial.pt.
25 controlled clinical trial.pt.
26 randomized.ab.
27 placebo.ab.
28 drug therapy.fs.
29 randomly.ab.
30 trial.ab.
31 groups.ab.
32 24 or 25 or 26 or 27 or 28 or 29 or 30 or 31
33 exp animals/ not humans.sh.
34 32 not 33

35 23 and 34
36 limit 35 to yr="2000 - 2019"

2) Embase (Ovid) Search Strategy

Database: Embase <1974 to 2021 February 12>
Search Strategy:
----
1 knee osteoarthritis/ or knee/
2 ("Knee Pain" or "Osteoarthritis Of Knee" or "knee Osteoarthritis" or "knee OA").ab,ti,tw.
3 (knee? adj (arthritis or osteoarthritis* or degeneration* or disease? or pain?)).ab,ti,tw.
4 ((radiographic* or symptomatic* or clinical*) adj1 knee osteoarthritis*).ab,ti,tw.
5 1 or 2 or 3 or 4
6 exercise/ or fitness/ or physical education/ or sport/ or exp kinesiotherapy/ or exp occupational therapy/ or exp physiotherapy/
7 (Physical* adj (fit or fitness or activit* or training? or therapy or therapies or endur* or life-style or lifestyle)).ab,ti,tw.
8 (exercise? or rehabilitation* or treatment* or sport* or walk* or bicycl* or (activ* adj (lifestyle or life-style))).ab,ti,tw.
9 ((resist* adj (exercise? or therapy or therapies or training?)) or occupational therap* or physiotherap*).ab,ti,tw.
10 6 or 7 or 8 or 9
11 body weight loss/ or body weight/ or exp caloric intake/ or exp obesity/ or diet/ or vegetarian diet/ or low carbohydrate diet/ or diet therapy/ or low calorie diet/
12 (dietary restriction? or meal replacement? or weight loss or intentional weight loss or hypocloric diet or obesity).ab,ti,tw.
13 (diet adj2 (therapy or therapies or treatment?)).ab,ti,tw.
14 11 or 12 or 13
15 psychotherapy/ or psychology/ or social psychology/ or cognitive behavioral therapy/ or relaxation training/ or leisure/ or guided imagery/ or hypnosis/ or exp behavior therapy/
16 (psychotherap* or psychological or psychology or psycho-education or cognitive therap* or behavio?ral therap* or relaxation or imagery or hypnosis or psychosocial or (adapt* adj behavio?r?) or (behavio?r? adj (therap* or intervention*))).ab,ti,tw.
17 15 or 16
18 patient education/ or health education/ or health promotion/ or primary prevention/
19 (promot* or educat* or program? or programme?).ab,ti,tw.
20 18 or 19
21 (multidisciplinar* or interdisciplinar* or multiprofessional* or multimodal*).ab,ti,tw.
22 14 or 17 or 20 or 21
23 5 and 10 and 22
24 crossover-procedure/ or double-blind procedure/ or randomized controlled trial/ or single-blind procedure/ or (random* or factorial* or crossover* or cross over* or placebo* or (doubl* adj blind*) or (singl* adj blind*) or assign* or allocat* or volunteer*).tw.
25 23 and 24
26 limit 25 to yr="2000 - 2021"

3) Cochrane Library Search Strategy

ID	Search
#1	MeSH descriptor: [Osteoarthritis, Knee] this term only
#2	MeSH descriptor: [Knee Joint] this term only
#3	("Knee Pain" or "Osteoarthritis Of Knee" or "knee Osteoarthritis" or "knee OA"):ti,ab,kw (Word variations have been searched)
#4	(knee? NEAR (arthritis or osteoarthritis* or degeneration* or disease? or pain?)):ti,ab,kw
#5	(((radiographic* or symptomatic* or clinical*) NEAR/1 "knee osteoarthritis*")):ti,ab,kw
#6	#1 OR #2 OR #3 OR #4 OR #5
#7	MeSH descriptor: [Exercise] this term only
#8	MeSH descriptor: [Physical Exertion] this term only
#9	MeSH descriptor: [Physical Fitness] this term only
#10	MeSH descriptor: [Sports] this term only
#11	MeSH descriptor: [Exercise Therapy] explode all trees
#12	MeSH descriptor: [Occupational Therapy] explode all trees
#13	MeSH descriptor: [Physical Therapy Specialty] explode all trees
#14	MeSH descriptor: [Physical Education and Training] this term only
#15	((Physical* NEAR (fit or fitness or activit* or training? or therapy or therapies or endur* or life-style or lifestyle))):ti,ab,kw
#16	((exercise? or rehabilitation* or treatment* or sport* or walk* or bicycl* or (activ* NEAR (lifestyle or life-style)))):ti,ab,kw
#17	(((resist* NEAR (exercise? or therapy or therapies or training?)) or "occupational therap*" or physiotherap*)):ti,ab,kw
#18	#7 OR #8 OR #9 OR #10 OR #11 OR #12 OR #13 OR #14 OR #15 OR #16 OR #17
#19	MeSH descriptor: [Weight Loss] this term only
#20	MeSH descriptor: [Body Weight] this term only
#21	MeSH descriptor: [Energy Intake] explode all trees
#22	MeSH descriptor: [Overweight] explode all trees
#23	MeSH descriptor: [Diet] this term only
#24	MeSH descriptor: [Diet, Vegetarian] this term only
#25	MeSH descriptor: [Diet, Carbohydrate-Restricted] this term only
#26	MeSH descriptor: [Diet Therapy] this term only
#27	MeSH descriptor: [Diet, Reducing] this term only
#28	("dietary restriction?" or "meal replacement?" or "weight loss" or "intentional weight loss" or "hypocloric diet" or obesity):ti,ab,kw
#29	((diet NEAR/2 (therapy or therapies or treatment?))):ti,ab,kw
#30	#19 OR #20 OR #21 OR #22 OR #23 OR #24 OR #25 OR #26 OR #27 OR #28 OR #29
#31	MeSH descriptor: [Psychotherapy] this term only
#32	MeSH descriptor: [Psychology] this term only
#33	MeSH descriptor: [Psychology, Educational] this term only
#34	MeSH descriptor: [Psychology, Social] this term only
#35	MeSH descriptor: [Cognitive Behavioral Therapy] this term only
#36	MeSH descriptor: [Relaxation Therapy] this term only
#37	MeSH descriptor: [Relaxation] this term only
#38	MeSH descriptor: [Imagery, Psychotherapy] this term only
#39	MeSH descriptor: [Hypnosis] this term only
#40	MeSH descriptor: [Behavior Therapy] explode all trees
#41	((psychotherap* or psychological or psychology or psycho-education or "cognitive therap*" or "behavio?ral therap*" or relaxation or imagery or hypnosis or psychosocial or (adapt* adj behavio?r?) or (behavio?r? NEAR (therap* or intervention*)))):ti,ab,kw
#42	#31 OR #32 OR #33 OR #34 OR #35 OR #36 OR #37 OR #38 OR #39 OR #40 OR #41

#43 MeSH descriptor: [Health Education] explode all trees
#44 MeSH descriptor: [Patient Education as Topic] this term only
#45 MeSH descriptor: [Health Promotion] this term only
#46 MeSH descriptor: [Primary Prevention] this term only
#47 ((promot* or educat* or program? or programme?)):ti,ab,kw
#48 #43 OR #44 OR #45 OR #46 OR #47
#49 ((multidisciplinar* or interdisciplinar* or multiprofessional* or multimodal*)):ti,ab,kw
#50 #30 OR #42 OR #48 OR #49
#51 #6 AND #18 AND #50 with Publication Year from 2000 to 2021, in Trials (Word variations have been searched

4) CINAHL Search Strategy

S1 (MH "Osteoarthritis, Knee") OR (MH "Knee Joint")
S2 TI ("Knee Pain" or "Osteoarthritis Of Knee" or "knee Osteoarthritis" or "knee OA") OR AB ("Knee Pain" or "Osteoarthritis Of Knee" or "knee Osteoarthritis" or "knee OA")
S3 TI (knee# N (arthritis or osteoarthritis* or degeneration* or disease# or pain#) OR AB (knee# N (arthritis or osteoarthritis* or degeneration* or disease# or pain#)
S4 TI ((radiographic* or symptomatic* or clinical*) N1 "knee osteoarthritis*") OR AB ((radiographic* or symptomatic* or clinical*) N1 "knee osteoarthritis*")
S5 S1 OR S2 OR S3 OR S4
S6 (MH "Exercise") OR (MH "Exertion") OR (MH "Physical Fitness") OR (MH "Physical Education and Training") OR (MH "Sports") OR (MH "Therapeutic Exercise+") OR (MH "Occupational Therapy+") OR (MH "Physical Therapy")
S7 TI (Physical* N (fit or fitness or activit* or training# or therapy or therapies or endur* or lifestyle or lifestyle)) OR AB (Physical* N (fit or fitness or activit* or training# or therapy or therapies or endur* or life-style or lifestyle))
S8 TI (exercise# or rehabilitation* or treatment* or sport* or walk* or bicycl* or (activ* N (lifestyle or life-style))) OR AB (exercise# or rehabilitation* or treatment* or sport* or walk* or bicycl* or (activ* N (lifestyle or life-style)))
S9 TI (((resist* N (exercise# or therapy or therapies or training#)) or "occupational therap*" or physiotherap*)) OR AB (((resist* N (exercise# or therapy or therapies or training#)) or "occupational therap*" or physiotherap*))
S10 S6 OR S7 OR S8 OR S9
S11 (MH "Weight Loss") OR (MH "Body Weight") OR (MH "Energy Intake") OR (MH "Obesity+") OR (MH "Diet") OR (MH "Vegetarianism") OR (MH "Diet, Low Carbohydrate") OR (MH "Diet Therapy") OR (MH "Diet, Reducing")
S12 TI ("dietary restriction#" or "meal replacement#" or "weight loss" or "intentional weight loss" or "hypocloric diet" or obesity) OR AB ("dietary restriction#" or "meal replacement#" or "weight loss" or "intentional weight loss" or "hypocloric diet" or obesity)
S13 TI ((diet N2 (therapy or therapies or treatment#))) OR AB ((diet N2 (therapy or therapies or treatment#)))
S14 S11 OR S12 OR S13
S15 (MH "Psychotherapy") OR (MH "Psychology") OR (MH "Psychology, Educational") OR (MH "Psychology, Social") OR (MH "Behavior Therapy+") OR (MH "Relaxation") OR (MH "Guided Imagery") OR (MH "Hypnosis")
S16 TI ((psychotherap* or psychological or psychology or psychoeducation or "cognitive therap*" or "behavio#ral therap*" or relaxation or imagery or hypnosis or psychosocial or (adapt* N behavio#r#) or (behavio#r# N (therap* or intervention*)))) OR AB ((psychotherap* or psychological or psychology or psychoeducation or "cognitive therap*" or "behavio#ral therap*" or relaxation or imagery or hypnosis or psychosocial or (adapt* N behavio#r#) or (behavio#r# N (therap* or intervention*))))

S17 S15 OR S16
S18 (MH "Health Education") OR (MH "Patient Education") OR (MH "Health Promotion")
S19 TI ((promot* or educat* or program# or programme#)) OR AB ((promot* or educat* or program# or programme#))
S20 S18 OR S19
S21 TI (multidisciplinar* or interdisciplinar* or multiprofessional* or multimodal*) OR AB (multidisciplinar* or interdisciplinar* or multiprofessional* or multimodal*)
S22 S14 OR S17 OR S20 OR S21
S23 S5 AND S10 AND S22
S24 (MH "randomized controlled trials")
S25 (MH "double-blind studies")
S26 (MH "single-blind studies")
S27 (MH "random assignment")
S28 (MH "pretest-posttest design")
S29 (MH "cluster sample")
S30 TI (randomised OR randomized)
S31 AB (random*)
S32 TI (trial)
S33 MH ("sample size") AND AB (assigned OR allocated OR control)
S34 MH ("placebos")
S35 PT ("randomized controlled trial")
S36 AB (control W5 group)
S37 MH ("crossover design") OR MH ("comparative studies")
S38 AB (cluster W3 RCT)
S39 MH ("animals+")
S40 MH ("animal studies")
S41 TI (animal model*)
S42 S39 OR S40 OR S41
S43 MH ("human")
S44 S42 NOT S43
S45 S24 OR S25 OR S26 OR S27 OR S28 OR S29 OR S30 OR S31 OR S32 OR S33 OR S34 OR S35 OR S36 OR S37 OR S38
S46 S45 NOT S44
S47 S23 AND S46
S48 S23 AND S46

5) PsycInfo Search Strategy

S1 DE "Knee"
S2 TI ("Knee Pain" or "Osteoarthritis Of Knee" or "knee Osteoarthritis" or "knee OA") OR AB ("Knee Pain" or "Osteoarthritis Of Knee" or "knee Osteoarthritis" or "knee OA")
S3 TI (knee# N (arthritis or osteoarthritis* or degeneration* or disease# or pain#) OR AB (knee# N (arthritis or osteoarthritis* or degeneration* or disease# or pain#)
S4 TI ((radiographic* or symptomatic* or clinical*) N1 "knee osteoarthritis*") OR AB ((radiographic* or symptomatic* or clinical*) N1 "knee osteoarthritis*")
S5 S1 OR S2 OR S3 OR S4
S6 (((((DE "Exercise") OR (DE "Physical Fitness")) OR (DE "Physical Education")) OR (DE "Sports")) OR (DE "Occupational Therapy")) OR (DE "Physical Therapy")
S7 TI (Physical* N (fit or fitness or activit* or training# or therapy or therapies or endur* or lifestyle or lifestyle)) OR AB (Physical* N (fit or fitness or activit* or training# or therapy or therapies or endur* or life-style or lifestyle))

S8 TI (exercise# or rehabilitation* or treatment* or sport* or walk* or bicycl* or (activ* N (lifestyle or life-style))) OR AB (exercise# or rehabilitation* or treatment* or sport* or walk* or bicycl* or (activ* N (lifestyle or life-style)))
S9 TI (((resist* N (exercise# or therapy or therapies or training#)) or "occupational therap*" or physiotherap*)) OR AB (((resist* N (exercise# or therapy or therapies or training#)) or "occupational therap*" or physiotherap*))
S10 S6 OR S7 OR S8 OR S9
S11 ((((DE "Weight Loss") OR (DE "Body Weight")) OR (DE "Overweight")) OR (DE "Obesity")) OR (DE "Diets")
S12 TI ("dietary restriction#" or "meal replacement#" or "weight loss" or "intentional weight loss" or "hypocloric diet" or obesity) OR AB ("dietary restriction#" or "meal replacement#" or "weight loss" or "intentional weight loss" or "hypocloric diet" or obesity)
S13 TI ((diet N2 (therapy or therapies or treatment#))) OR AB ((diet N2 (therapy or therapies or treatment#)))
S14 S11 OR S12 OR S13
S15 (((((((((DE "Psychotherapy") OR (DE "Psychology")) OR (DE "Educational Psychology")) OR (DE "Social Psychology")) OR (DE "Cognitive Behavior Therapy")) OR (DE "Relaxation Therapy")) OR (DE "Relaxation")) OR (DE "Imagery")) OR (DE "Hypnosis")) OR (DE "Behavior Therapy" OR DE "Aversion Therapy" OR DE "Conversion Therapy" OR DE "Dialectical Behavior Therapy" OR DE "Exposure Therapy" OR DE "Implosive Therapy" OR DE "Reciprocal Inhibition Therapy" OR DE "Response Cost" OR DE "Systematic Desensitization Therapy")
S16 TI ((psychotherap* or psychological or psychology or psychoeducation or "cognitive therap*" or "behavio#ral therap*" or relaxation or imagery or hypnosis or psychosocial or (adapt* N behavio#r#) or (behavio#r# N (therap* or intervention*)))) OR AB ((psychotherap* or psychological or psychology or psychoeducation or "cognitive therap*" or "behavio#ral therap*" or relaxation or imagery or hypnosis or psychosocial or (adapt* N behavio#r#) or (behavio#r# N (therap* or intervention*))))
S17 S15 OR S16
S18 ((DE "Health Education") OR (DE "Client Education")) OR (DE "Health Promotion")
S19 TI ((promot* or educat* or program# or programme#)) OR AB ((promot* or educat* or program# or programme#))
S20 S18 OR S19
S21 TI (multidisciplinar* or interdisciplinar* or multiprofessional* or multimodal*) OR AB (multidisciplinar* or interdisciplinar* or multiprofessional* or multimodal*)
S22 S14 OR S17 OR S20 OR S21
S23 S5 AND S10 AND S22
S24 (((DE "Treatment Effectiveness Evaluation") OR (DE "Treatment Outcomes" OR DE "Psychotherapeutic Outcomes" OR DE "Side Effects (Treatment)" OR DE "Treatment Compliance" OR DE "Treatment Duration" OR DE "Treatment Refusal" OR DE "Treatment Termination" OR DE "Treatment Withholding")) OR (DE "Placebo")) OR (DE "Followup Studies")
S25 TX placebo* OR random* OR "comparative stud*" OR clinical N3 trial* OR research N3 design OR evaluat* N3 stud* OR prospectiv* N3 stud* OR (singl* OR doubl* OR trebl* OR tripl*) N3 (blind* OR mask*)
S26 S24 OR S25
S27 S23 AND S26
S28 S23 AND S26
