# Supplementary material for: Complex Lifestyle and Psychological Intervention in Knee Osteoarthritis: Scoping Review of Randomized Controlled Trials
Source: Int J Environ Res Public Health. 2021 Dec 3;18(23):12757. doi: 10.3390/ijerph182312757 (PMC8657138; doi:10.3390/ijerph182312757)
Supplement: Supplementary file 1 [file ijerph-18-12757-s001.zip › ijerph-1403346-supplementary/IJERPH Supplementary S2.pdf]

Supplementary S2

Title	Author, Year	Inclusion Criteria *beyond OA knee	Intervention					Control Arm	Outcome Measures		Study Size	Results (Intervention/Control/No difference) *based on primary outcome	Process Evaluation	Economic Evaluation
			Supervised Physical Intervention	Supervised Dietary Intervention	Self-Management/Education	Psychological	TIDieR score		Primary	Secondary				
Evaluation of the Interaction Model of Client Health Behavior-based multifaceted intervention on patient activation and osteoarthritis symptoms	Ahn & Ham, 2020	Nil	Group E1: muscle strengthening Group E2: walking exercise 4 individual sessions and 4 group sessions	x	Health education	Counselling	67%	Usual care (treatment and prescription with minimal health education and counseling)	PAM	WOMAC, HAQDS, CES-D	90	Intervention	No	No
Efficacy of an exercise program combined with lifestyle education in patients with knee osteoarthritis	Alfieri et al, 2020	Nil	Warm-up, flexibility, muscle strengthening, balance and proprioception 2 times/week for 8 weeks	x	8 sessions of lectures and discussion on disease self-management and healthy lifestyle education	X	50%	Exercise alone	VAS	lifestyle symptoms, WOMAC, PPT	39	Intervention	No	No
Stepped Exercise Program for Patients With Knee Osteoarthritis : A Randomized Controlled Trial	Allen et al, 2020	Nil	x	x	Stepped exercise program Step 1: internet-based exercise training program Step 2: adds telephone-based coaching Step 3: adds a series of in-person physical therapy visits	x	58%	Arthritis Education with low literacy educational materials via mail every 2 weeks	WOMAC at 9 months	Physical perfomance (chair stand, face-paced walk) at 9 months	345	Intervention	No	No
Effects of rural community-based integrated exercise and health education programs on the mobility function of older adults with knee osteoarthritis	Oh, Kim, Bae & Lim, 2020	Nil	Self directed home-based resistance training for 2 or 3 days/week for 5 months	x	Modified health education program emphasizing regular physical exercise, 50mins once a month for 5 months	x	75%	Education alone	Mobility function (timed chair stand, up and go, gait speed, knee extensor strength)	WOMAC, SARC-F, SOF	60	Intervention	Adherence (logbook)	No
Limited effect of a self-management exercise program added to spa therapy for increasing physical activity in patients with knee osteoarthritis: a quasi-randomized controlled trial	Gay et al, 2019	Nil	Spa sessions over 3 weeks by PT	x	5 Self-management sessions on exercise	x	83%	Written information booklet with spa therapy	Physical activity (IPAQ) at 3 months	Self-Efficacy (ASES), WOMAC, Anxiety and Depression (HADS), Fears and beliefs (KOFBeQ), Pain	123	Intervention	Adherence (practice and frequency of booklet exercise at 3 months)	No
Effect of a Positive Psychological Intervention on Pain and Functional Difficulty Among Adults with Osteoarthritis: A Randomized Clinical Trial	Hausmann et al, 2018	Nil	x	x	x	Staying positive with arthritis, 6-week program to build positive psychological skills e.g. gratitude, kindness by staff trained by psychologist	75%	Affectively neutral but structurally similar program	WOMAC at 1,3,6 months	Affect Balance and Life Satisfaction	360	No difference	Adherence (number of weekly calls completed and number of correctly identified activities that were partially or entirely completed)	No
Effects of a 12-Week Digital Care Program for Chronic Knee Pain on Pain, Mobility, and Surgery Risk: Randomized Controlled Trial.	Mecklenburg et al, 2018	Nil	x	x	Hinge Health digital program, 12 weeks, remotely delivered, sensor guided exercise therapy, education, CBT, weight loss, psychosocial support through coaching	x	75%	Usual Care (Education)	KOOS at 11 weeks	Knee pain, Knee stiffness, Surgery interest, Ability to self-manage	162	Intervention	No	No
Physical therapy vs internet-based exercise training for patients with knee osteoarthritis: results of a randomized controlled trial	Allen at al, 2018	Nil	x	x	IBET program (tailored algorithm exercise and progression, video display of exercises, automated reminders, progress tracking)	x	75%	No intervention	WOMAC at 4 months, 12 months	Physical Performance, Depression (PHQ), KOOS, PROMIS sleep, Fear of movement, Physical activity (PASE)	350	No difference	No	No
A randomized controlled trial of a combined self-management and exercise intervention for elderly people with osteoarthritis of the knee: the PLE2NO program	Marconcin et al, 2018	Nil	Exercise for 12 weeks by PT	x	PLE2NO program, group-based, 12 weeks	x	75%	Usual Care (Education, glucosamine)	KOOS, Self-Efficacy, Physical Performance (Strength, Cardiorespiratory endurance, flexibility, grip strength) at 3 months	EQ-5D, Physical Performance (gait speed, balance, mobility)	67	Intervention	No	No

Supplementary S2

Telephone-based weight loss support for patients with knee osteoarthritis: a pragmatic randomised controlled trial	O'Brien et al, 2018	Nil	x	x	NSW Get Healthy Information and Coaching Service (GHS), 10 telephonic sessions over 6 months by trained allied healthcare professionals focusing on dietary and physical activity lifestyle goals	x	75%	Usual Care (Education)	Pain intensity at 2,6,10,14,18,22,26 weeks	WOMAC, SF12, Diet change (FFQ), Weight, Physical activity	120	No difference	Adherence (telephone calls)	Yes
Internet Cognitive-Behavioural Therapy for Depression in Older Adults with Knee Osteoarthritis: A Randomized Controlled Trial	O'Moore et al, 2018	Major Depressive Disorder	x	x	x	iCBT Sadness Program, 6 lessons, cognitive behavioural therapy, telephonic follow up if deterioration	75%	Usual Care (Education)	Depression (PHQ), Psychological distress (K-10) at 4,11,24 weeks	SF-12, WOMAC, Self-Efficacy (ASES)	100	Intervention	Adherence (lesson completion)	No
Efficacy of tele-rehabilitation compared with office-based physical therapy in patients with knee osteoarthritis: a randomized clinical trial	Azma et al, 2018	Nil	x	x	Telerehab for exercise, 6 weeks with weekly telephonic follow up, educational pamphlet, logbook	x	58%	Office Based PT (OBPT)	KOOS, WOMAC at 6 weeks, 1 month and 6 months	KOOS, WOMAC subscales	54	No difference	No	No
Telephone Coaching to Enhance a Home-Based Physical Activity Program for Knee Osteoarthritis: A Randomized Clinical Trial	Bennell et al, 2017	Nil	Individualized PT session over 6 months	x	Telephonic Coaching using HealthChange methodology by health coach	x	75%	PT alone	Pain, WOMAC at 6 months	Physical activity (PASE, accelerometer), Global change, QOL, Psychological, Health service utilization	168	Intervention	Adherence (completion of home exercise)	No
Effectiveness of an Internet-Delivered Exercise and Pain-Coping Skills Training Intervention for Persons with Chronic Knee Pain: A Randomized Trial	Bennell et al, 2017	Nil	7 Skype PT sessions	x	3 Internet delivered education sessions (exercise, physical activity, pain, emotion, diet, meds)	8 Internet delivered pain coping skills training sessions	92%	Internet based Educational Material	Pain on walking and WOMAC at 3 months	Pain, QOL, Global change, Self-efficacy (ASES), Coping (CSQ), Pain catastrophizing (PCS)	148	Intervention	Adherence (number of Skype physiotherapy sessions attended, number of pain-COACH sessions completed)	No
Efficacy of Tailored Exercise Therapy on Physical Functioning in Patients with Knee Osteoarthritis and Comorbidity: A Randomized Controlled Trial	de Rooij et al, 2017	Presence of at least 1 target comorbidity	Comorbidity-related individualized exercise over 20 weeks	x	Education on exercise adaption, weight management, fear of exertion	x	83%	Usual care (Education)	WOMAC, 6 min walk test at 10,20,32 weeks	Knee pain, WOMAC subscale, SF-36, Physical activity (LAPAQ)	126	Intervention	Compliance	No
A walking program for people with severe knee osteoarthritis did not reduce pain but may have benefits for cardiovascular health: a phase II randomised controlled trial	Wallis et al, 2017	History of cardiovascular disease	Exercise with PT	x	Walking program over 12 weeks, planning session with PT, pedometer, Telephonic/SMS follow-up, Social support	x	92%	Usual Care (Education)	Knee pain at 12 weeks	Physical activity, resting blood pressure, BMI, waist circumference, total cholesterol level, WOMAC, QOL, Physical Performance (40m walk, 30s chair stand)	46	No difference	Adherence (logbook)	No
Physical Therapist-Delivered Pain Coping Skills Training and Exercise for Knee Osteoarthritis: randomized Controlled Trial	Bennell et al, 2016	Nil	Exercise class with PT over 12 weeks	x	x	Pain Coping skill training by PT (PCST)	67%	PCST or Exercise alone	Knee pain, WOMAC at 12, 32, 52 weeks	Pain, Global change, Physical performance, Psychological health, Physical activity, QOL	222	Intervention	Adherence (telephone call from physical therapists at week 22, 38 and 46)	Yes
The Effect of Multifactorial Intervention Programs on Health Behaviour and Symptom Control Among Community-Dwelling Overweight Older Adults With Knee Osteoarthritis	Saraboon et al, 2015	Nil	x	x	MUFIP program (education, dietary change, exercise), home visits	x	75%	Usual Care (Education)	Health behaviour and symptoms	Knee Pain, OA knowledge test, Pain score, Health Behavioural Questionnaire, Physical Performance, ROM, Weight	80	Intervention	No	No
Cognitive-behavioural therapy for insomnia in knee osteoarthritis: a randomized, double-blind, active placebo-controlled clinical trial	Smith et al, 2015	Insomnia	x	x	x	Cognitive Behavioural Therapy for Insomnia by PSY	92%	Behavioural desensitization	Wake After Sleep Onset, Pain Intensity Index at 3,6 months	Sleep diary, Actigraphy measure of sleep continuity, Insomnia severity index, WOMAC, Conditioned pain	100	Intervention	Adherence (questionnaire before each treatment on how closely and frequently patients follow	No

Supplementary S2

										modulation, temporal summation			interventionist's prescription)	
Effect of weight maintenance on symptoms of knee osteoarthritis in obese patients: a twelve-month randomized controlled trial	Christensen et al, 2015	BMI>30	Exercise program with PT x 52 weeks	Dietician sessions x 52 weeks	x	x	67%	Dietary sessions x 16 weeks	Pain Score, Weight, OMERACT-OARSI responder criteria (pain, disability, global) at 1 year	KOOS, SF-36, Gait speed, DEXA, X-rays	192	Intervention	Compliance	No
Effectiveness of a cognitive-behavioural group intervention for knee osteoarthritis pain: a randomized controlled trial	Helminen et al, 2015	Nil	x	x	x	CBT group 7-13 pax session weekly x 6 weeks by PSY and PT (knowledge, problem solving, skills)	83%	Usual Care (Education, medication)	WOMAC at 3, 12 months	Pain Score, WOMAC subscale, QOL (RAND-36, 15D), Use of analgesia, Life Satisfaction, Psychological (Self-Efficacy, Coherence, Pain Catastrophizing, Kinesiophobia, depression, anxiety) Global assessment	111	No difference	No	No
The efficacy of 12 weeks non-surgical treatment for patients not eligible for total knee replacement: a randomized controlled trial with 1-year follow-up	Skou et al, 2015	Nil	NEMEX exercise over 12 weeks, transition 8 weeks to home, telephonic follow up	BMI>25, 4 session over 12 weeks with dietician, telephonic follow up	MEDIC treatment Education 2 x session focus on self help	x	83%	Usual Care (Education)	KOOS at 12 months	KOOS subscale, Weight change, Analgesia use, Physical performance (20m walk, TUG)	100	Intervention	No	No
Randomized clinical trial of group Vs. Individual physical therapy for knee osteoarthritis	Allen et al, 2014	Nil	Group PT over 6 weeks	x	Education on pacing daily activities, protecting joints, home exercise, barriers	x	83%	Individual PT	WOMAC at 12,24 weeks	Short Physical Performance Battery (SPPB)	320	No difference	Adherence (periodic monitoring by physical therapist)	No
Effects of Intensive Diet and Exercise on Knee Joint Loads, Inflammation, and Clinical Outcomes Among Overweight and Obese Adults With Knee Osteoarthritis: The IDEA Randomized Clinical Trial	Messier et al, 2013	BMI 27-41	Exercise program with PT x 18 months	Nutritionist sessions x 18 months	Adherence data guided, behavioural toolbox (additional counselling, social support, incentives)	PT and nutritionist trained in behavioural techniques CBT and group dynamics	67%	Dietary or exercise intervention individually	Knee Compressive Forces at 6, 18 months	IL-6, WOMAC, SF-36, Physical Performance (gait speed, 6m walk test), BMI, DEXA	399	Intervention	Adherence (daily logs)	Yes
Pain coping skills training and lifestyle behavioural weight management in patients with knee osteoarthritis: a randomized controlled study	Somers et al, 2012	Nil	Group Exercise with PT	x	Behavioural Weight Management (BWM) - LEARN (lifestyle, exercise, attitudes, relationships, nutrition), Group session x 24 weeks, PSY, telephonic follow up	Pain coping skill training (PCST), Group session over 24 weeks, PSY	83%	BWM alone or PCST alone or Usual care	AIMS, WOMAC	Gait speed, pain catastrophizing, self-efficacy, BMI	232	Intervention	Adherence (phone call)	No
The implementation of a community-based aerobic walking program for mild to moderate knee osteoarthritis: a knowledge translation randomized controlled trial: part II: clinical outcomes	Brosseau et al, 2012	Nil	Supervised Community-based aerobic walking program (SCAWP)	x	Behavioural Intervention (coaching, goal setting, education, social/peer support)	x	75%	Usual Care (Education)	QoL	AIMS, SF36, WOMAC, Physical Performance (6m walk test, TUG), Physical activity at 6, 12 months	222	No difference	Adherence (number of attended walking sessions, log books)	No
A randomised controlled trial of a self-management education program for osteoarthritis of the knee delivered by health care professionals	Coleman et al, 2012	Nil	x	x	OAK program, group session over 6 weeks, Nurses and PT, Social cognitive therapy/CBT (medication counselling, exercise, nutrition, fall prevention, environmental risk)	x	67%	6 months wait prior to starting the program	WOMAC, SF-36 at 8 weeks, 6 months	Pain Score, Physical Performance (TCU, ROM, Quadricep and Hamstring strength)	146	Intervention	No	No
Pharmacist-initiated intervention trial in osteoarthritis: a multidisciplinary intervention for knee osteoarthritis	Marra et al, 2012	Nil	Personalized exercise by PT over 6 weeks	x	Pharmacist initiated education, medication review, referral to PT, primary physician update	x	67%	Usual Care (Education)	Quality of care measures at 6 months	WOMAC, Lower Extremity Function Scale, Paper Adaptive Test-5D, Health Utilities Index Mark 3	139	Intervention	No	No

Supplementary S2

Intensive lifestyle intervention improves physical function among obese adults with knee pain: findings from the Look AHEAD trial	Foy et al, 2011	Type 2 DM, BMI>25	x	x	Intensive Lifestyle Intervention (ILI) Group and individual over 6 months, Lifestyle counseller, toolbox of behavioural strategies, pharmacotherapy with orlistat, focus on dietary change and increasing physical activity	x	75%	Diabetes Support and Education (DSE)	WOMAC at 12 months	Nil	2203	Intervention	No	No
Progressive resistance training improves overall physical activity levels in patients with early osteoarthritis of the knee: a randomized controlled trial	Farr et al, 2010	Nil	Resistance Training with PT	x	Education on self-efficacy (exercise, nutrition, analgesia, stress management, coping mechanism)	x	58%	Exercise or self-management alone	WOMAC, Physical Activity (MTI Actigraph accelerometer, ACLS) at 3,9 months	Nil	293	Intervention	No	No
A comparison of strength-training, self-management and the combination for early osteoarthritis of the knee	McKnight et al, 2010	Nil	Strength training with PT over 9 months	x	Self-management over 12 weeks on coping and self-efficacy skills by program manager, telephonic follow up	x	67%	Exercise or self-management alone	WOMAC, SF-36, Physical Performance (Leg Press, ROM, ERGOS work stimulator, TUG, stair climbing) at 9,24 months	VASl	273	No difference	No	No
Effects of dietary intervention and quadriceps strengthening exercises on pain and function in overweight people with knee pain: randomised controlled trial	Jenkinson et al, 2009	BMI>28	x	EPIC food diary intervention over 18 months, dietician, home visit	Exercise taught by dietician to be done unsupervised	x	83%	Exercise or Dietary intervention alone	WOMAC at 24 months	WOMAC subscales, Hospital Anxiety and Depression Scale, SF-36	389	No difference	Compliance (exercise diaries)	Yes
ARTIST (osteoarthritis intervention standardized) study of standardised consultation versus usual care for patients with osteoarthritis of the knee in primary care in France: Pragmatic randomised controlled trial	Ravaud et al, 2009	Nil	x	x	3 goal orientated session with rheumatologist over 1 month focus on education, exercise and weight loss	x	75%	Usual Care (Education)	Weight, Physical Activity (Baecke index) at 4, 12 months	WOMAC, SF-12	336	Intervention	No	No
Effects of a self-management arthritis programme with an added exercise component for osteoarthritic knee: randomized controlled trial	Yip et al, 2007	Nil	x	x	Modified ASMP (Stanford Uni) and goal directed exercise, group approach focus on self-efficacy and behaviour change, nurses, exercise action plan with pedometer	x	83%	Usual Care	Self-efficacy (ASE), Use of self-management techniques, Pain Score, Health Assessment Questionnaire (HAQ) at 16 weeks	Nil	120	Intervention	No	No
Physical exercise and comorbidity. Results from the Fitness and Arthritis in Seniors Trial (FAST)	Mangani et al, 2006	2006	Aerobic Exercise (AE) or Weight Training (WT) over 3 months, home visit and telephonic follow up	x	Health Education (HE) sessions over 3 months, nurse, telephonic follow up	x	75%	HE vs AE vs WT alone	Physical Performance (6min walk test), Pain score, Disability	Nil	475	Intervention	Adherence (attendance in exercise sessions)	No
Effects of spouse-assisted coping skills training and exercise training in patients with osteoarthritic knee pain: a randomized controlled study	Keefe et al, 2004	2004	Exercise Training by PT	x	x	Spouse assisted pain coping skills training by PSY	58%	Usual Care	Aerobic fitness and strength, Pain coping (CSQ)	Self-Efficacy (ASE), Marital adjustment (Dyadic Adjustment Scale), AIMS	72	Intervention	No	No
Can a nurse-directed intervention reduce the exposure of patients with knee osteoarthritis to nonsteroidal anti-inflammatory drugs?	Mazzuca et al, 2004	2004	x	x	18 weeks nurse-led clinical algorithm (exercise, joint protection, weight loss, shoe and walking aid advice, analgesia use)	x	58%	Usual Care	WOMAC at 3,6,12 months	Nil	186	Intervention	No	No
Diet-induced weight loss, exercise, and chronic inflammation in older, obese adults: a randomized controlled clinical trial	Nicklas et al, 2004	2004	Exercise (Aerobic, Resistance) over 18 months, home/facility based	Group and individual sessions over 18 months, Dietician, telephonic follow ups, newsletters	X	x	83%	Exercise vs Diet alone	Blood test (CRP, IL6, TNF-alpha) at 6, 18 months	Nil	300	Control (Diet)	Adherence (attendance at scheduled sessions and monthly weight assessments)	No
